# Supplementary material for: Pharmacists’ Perceptions and Drivers of Immunization Practices for COVID-19 Vaccines: Results of a Nationwide Survey Prior to COVID-19 Vaccine Emergency Use Authorization
Source: Pharmacy (Basel). 2021 Jul 26;9(3):131. doi: 10.3390/pharmacy9030131 (PMC8396166; doi:10.3390/pharmacy9030131)
Supplement: Supplementary file 1 [file pharmacy-09-00131-s001.zip › pharmacy-1304207-supplementary.pdf]

Supplementary Appendix

Table S1: Survey Questionnaire

Demographic Survey Questionnaire		
**Category**	**Idea Behind Question**	**Framed Question**
**Consent**	Consent form	**Question 1:** **If answer is (2) - participant will be unable to complete survey**<br>**By beginning this research, I acknowledge that I am 18 years of age or older and have read and understand the information. I agree to take part in the research, with the knowledge that I am free to withdraw my participation in the research without penalty.**<br>(1) I Agree<br>(2) I Do Not Agree
**Inclusion Criteria**	Inclusion criteria	**Question 2:** **If answer is (1) - participant directed to question 3. If answer is (2) - participant will be unable to complete survey**<br>**Are you currently a licensed or practicing pharmacist in the United States?**<br>(1) Yes<br>(2) No
		**Question 3:**<br>**What state within the U.S. do you practice pharmacy in?** **If participants answered "I do not reside in the United States," they were unable to complete survey**<br><br>(Drop down with all 50 states, District of Columbia, Puerto Rico, and "I do not reside in the United States")
**Personal history**	Gender/Sex	**Question 4:**<br>**Which gender do you currently identify as?**<br>(1) Male<br>(2) Female<br>(3) Transgender man/female-to-male<br>(4) Transgender woman/ male-to-female<br>(5) Non-binary, genderqueer, or gender nonconforming<br>(6) Other (specify) ________<br>(7) Prefer not to answer
	Age	**Question 5 :**<br>**What is your age?**<br>(Free text – numerical values only)
	Race/Ethnicity	**Question 6:**<br>**Which of the following categories describes your race?**<br>(1) White<br>(2) Black<br>(3) Hispanic or Latinx<br>(4) American Indian<br>(5) Alaska Native<br>(6) Asian<br>(7) Native Hawaiian<br>(8) Pacific Islander<br>(9) Other (specify)<br>(10) Don't know<br>(11) Prefer not to answer
	Are they considered at increased risk of severe illness from COVID-19 (According to CDC)?	**Question 7:**<br>**Which, if any, of the following medical conditions do you have? (*Please select all that apply)***<br><br>1) Cancer

		2) Chronic kidney disease<br>3) Chronic obstructive pulmonary disease<br>4) Immunocompromised state from solid organ transplant<br>5) Obesity (BMI ≥30)<br>6) Serious heart condition, such as heart failure, coronary artery disease, or cardiomyopathies<br>7) Sickle cell disease<br>8) Type 2 diabetes mellitus<br>9) Asthma (moderate to severe)<br>10) Cerebrovascular disease<br>11) Cystic fibrosis<br>12) Immunocompromised state from bone marrow transplant, immune deficiencies, HIV, use of corticosteroids or other immune weakening medicines<br>13) Neurologic conditions, such as dementia<br>14) Liver disease<br>15) Pregnancy<br>16) Pulmonary fibrosis<br>17) Smoking<br>18) Thalassemia<br>19) Type 1 diabetes<br>20) None of the above
**Pharmacy Practice**	Years of practice	**Question 8:**<br>**How many years have you been a practicing pharmacist?**<br>(Free text – numerical values only)
	Pharmacy practice	**Question 9:**<br>**What pharmacy setting do you <u>primarily</u> work in?**<br>(1) Hospital pharmacy<br>(2) Outpatient clinic<br>(3) Community pharmacy<br>(4) Pharmaceutical industry<br>(5) Managed care<br>(6) Academia<br>(7) Other ________<br><br>**Question 10:**<br>**Do you work in a specialized therapeutic area? If so, what is it?**<br>(1) Ambulatory care<br>(2) Infectious Disease/HIV<br>(3) Transplant<br>(4) Oncology<br>(5) Neurology<br>(6) Anticoagulation/Cardiology<br>(7) Critical Care<br>(8) Emergency Medicine<br>(9) Pediatrics<br>(10) Geriatrics<br>(11) Internal medicine<br>(12) Other _________<br>(13) Not applicable
	Post-doctoral training	**Question 11:** ***If answered (1), (2), (3) are selected proceed to question 12. If answered (4), skip to question 13.***<br>**Which if the following post-doctoral training, if any, have you completed or are working on completing?**<br>(1) Residency<br>(2) Fellowship<br>(3) Both residency and fellowship<br>(4) None<br>**Question 12:**

		**If a postdoctoral training was complete, what was it completed in?**<br>(1) Ambulatory care<br>(2) Infectious Disease/HIV<br>(3) Transplant<br>(4) Oncology<br>(5) Neurology<br>(6) Anticoagulation/Cardiology<br>(7) Critical Care<br>(8) Emergency Medicine<br>(9) Pediatrics<br>(10) Geriatrics<br>(11) Internal medicine<br>(12) Other ________
**Comfortability/ Knowledge of COVID-19 /Perceived risk and severity**	Adapted from CDC National Immunization Survey: 2009 H1N1 Knowledge, attitudes, practice-<br>Question 13: Perceived risk (susceptibility)<br><br>Does someone who has personally had COVID-19 feel that they are already protected and therefore do not need the vaccine?<br><br>Adapted from H1N1 Alkuwari et. al Qatar survey study<br>Question 15: Does the pharmacist perceive COVID-19 as a severe/serious disease that can impact their daily life.<br><br>Question 16:<br>Influenza study by Petek et. al showed that familiarity with influenza disease did not impact the decision to vaccinate for influenza - will the same hold true for COVID-19? Rapidly changing knowledge of the virus/disease state, will this influence the pharmacist's view of credibility of a new vaccine?	**Please respond with how much you agree with the following statements.**<br><br>**Question 13:** ***If (1) is selected, skip to question 16. If (2) is selected proceed to question 14.***<br>**Have you had a confirmed diagnosis of COVID-19?**<br>(1) Yes<br>(2) No<br><br>**Please respond with how much you agree with the following statements.**<br><br>**Question 14:**<br>**I am concerned about becoming infected with SARS-CoV-2.**<br>(1) Strongly disagree<br>(2) Disagree<br>(3) Neutral/Don't know<br>(4) Agree<br>(5) Strongly agree<br><br>**Question 15:**<br>**My daily work will be affected if I get COVID-19.**<br>(1) Strongly disagree<br>(2) Disagree<br>(3) Neutral/Don't know<br>(4) Agree<br>(5) Strongly agree<br><br>**Question 16:**<br>**I have been following the news and scientific literature regarding current COVID-19 vaccine trials.**<br>(1) Strongly disagree<br>(2) Disagree<br>(3) Neither agree nor disagree<br>(4) Agree<br>(5) Strongly agree

	Based on a statement from the WHO That says COVID-19 pandemic is a wave, not seasonal (Released July 28th, 2020)<br><br>Do pharmacists believe that COVID-19 is a temporary/seasonal problem or do they believe it is wave that can be controlled by human action - will this belief impact them to get the vaccine?<br><br>https://www.reuters.com/article/us-health-coronavirus-who/who-says-covid-19-pandemic-is-one-big-wave-not-seasonal-idUSKCN24T16U	**Question 17:**<br>**I believe COVID-19 will be a seasonal virus.**<br>(1) Strongly disagree<br>(2) Disagree<br>(3) Neutral/Don't know<br>(4) Agree<br>(5) Strongly agree<br><br>**Question 18:**<br>**COVID-19 can occur in any season and is dependent on human control to slow the spread.**<br>(1) Strongly disagree<br>(2) Disagree<br>(3) Neutral/Don't know<br>(4) Agree<br>(5) Strongly agree
**Personal Experience with COVID-19**	To see if pharmacist who care for COVID-19 vaccine patients are more or less likely to get the vaccine (include community pharmacy with covid-19 testing sites, clinical settings)<br><br>CDC Interim Guidance for Critical Workers Who Have Had Exposure https://www.cdc.gov/coronavirus/2019-ncov/community/critical-workers/implementing-safety-practices.html<br><br>https://www.cdc.gov/coronavirus/2019-ncov/if-you-are-sick/quarantine.html	**Question 19:**<br>**Have you worked in close contact or cared for a person who had COVID-19?**<br>(1) Yes<br>(2) No<br>(3) Unsure<br><br>**Question 20:**<br>**The CDC defines COVID-19 exposure as having EITHER of the following**<br>• **Close contact within 6 feet for 15 minutes of an individual with confirmed or suspected COVID-19**<br>• **Close contact with the individual 48 hours prior to them becoming symptomatic**<br><br>**Based on this definition, have you been exposed to a person with COVID-19?**<br>(1) Yes<br>(2) No<br>(3) Unsure
	To gauge the impact of a personal loss on someone's willingness	**Question 21:**<br>**Has a loved one fallen critically ill or passed away due to COVID-19?**<br><br>(1) Yes<br>(2) No

COVID-19 Vaccine Related Survey Questionnaire

Category	Idea behind the question	Framed Question
**Decisions to receive and recommend**<br><br><br><br>*Includes*<br><br>*Time from approval to vaccination*<br><br>+<br>*Barriers/ Reasons to receive/ recommend*	**Willingness to Receive**<br><br>Question 22:<br>Are pharmacist likely to receive the vaccine $\leq$1 year since approval<br><br>Question 23:<br>Are pharmacists likely to receive a vaccine >1 year from approval?<br><br><br>Adapted from CDC National Immunization Survey: 2009 H1N1 Knowledge, attitudes, practice<br><br><br><br><br>Question 24: Barriers to receive<br><br>Question 25: Reasons to receive	**Question 22:**<br>**If a COVID-19 vaccine was deemed safe and effective by the FDA, how likely would you be to *get vaccinated* LESS THAN OR EQUAL to 1 year from the time of vaccine approval?**<br>(1) Extremely unlikely<br>(2) Somewhat unlikely<br>(3) Neither likely nor unlikely<br>(4) Somewhat likely<br>(5) Extremely likely<br><br>**Question 23:**<br>**If a COVID-19 vaccine was deemed safe and effective by the FDA, how likely would you be to get vaccinated GREATER THAN 1 year after vaccine approval?**<br>(1) Extremely unlikely<br>(2) Somewhat unlikely<br>(3) Neither likely nor unlikely<br>(4) Somewhat likely<br>(5) Extremely likely<br><br>**Question 24:** *Question will only appear to those who answer "extremely unlikely", "somewhat unlikely", "neither likely nor unlikely" to **Question 22 OR 23** (revolved around decision about getting vaccinated regardless of time frame of vaccine approval).*<br><br>**Which of the following would be the reason for your decision to delay or refuse the vaccine for yourself? Please select all that apply.**<br>(1) Concerns about side effects or sickness<br>(2) Think the vaccine does not work<br>(3) Vaccination is not needed or not wanted<br>(4) Because I already had COVID-19<br>(5) Not in a high-risk or priority group<br>(6) Limited contact with high risk patients<br>(7) COVID-19 is a non-severe or temporary issue<br>(8) Rapidly changing real world data during pandemic<br>(9) The ability to social distance and wear a mask provides sufficient protection<br>(10) Other _____________<br><br>**Question 25:** *Question will only appear for those who are "somewhat likely," or "extremely likely," to get vaccinated in **Questions 22 OR 23** (revolved around decision about getting vaccinated regardless of timeframe of vaccine approval)*<br><br>**If you were to receive the COVID-19 vaccine for yourself, which of the following would be reasons for your decision? Please select all that apply.**<br>(1) Wish to protect self<br>(2) Being a healthcare worker<br>(3) Avoid transmitting COVID-19 to my family<br>(4) Avoid transmitting COVID-19 to my patients<br>(5) Workplace mandate<br>(6) Have a medical condition that puts me at increased risk for COVID-19<br>(7) Recommendations of other medical professionals<br>(8) Recommendations of friends and family<br>(9) Recommendations of government health agencies (CDC, FDA, NIH, IDSA etc.)<br>(10) Recommendations of political leaders<br>(11) Other ______________

<table>
<tr>
<td></td>
<td><u>Willingness to Recommend</u>

Question 26:
Are pharmacist likely to recommend the vaccine ≤ 1 year since approval

Adapted from CDC National Immunization Survey: 2009 H1N1 Knowledge, attitudes, practice

Question 27
Are pharmacist likely to recommend the vaccine
> 1 year since approval:

Question 28:
Barriers to recommend

Question 29:
Reasons to recommend</td>
<td>Question 26:
If a COVID-19 vaccine was deemed safe and effective by the FDA, how likely are you to <u>recommend</u> the COVID-19 vaccine to patients LESS THAN OR EQUAL 1 year from the time of vaccine approval?
(1) Extremely unlikely
(2) Somewhat unlikely
(3) Neither likely nor unlikely
(4) Somewhat likely
(5) Extremely likely

Question 27:
If a COVID-19 vaccine was deemed safe and effective by the FDA, how likely are you to <u>recommend</u> the COVID-19 vaccine to your patients GREATER THAN 1 year after vaccine approval?
(1) Extremely unlikely
(2) Somewhat unlikely
(3) Neither likely nor unlikely
(4) Somewhat likely
(5) Extremely likely

Question 28: Question will only appear to those who answer “extremely unlikely”, “somewhat unlikely”, “neither likely nor unlikely” to Question 26 <u>OR</u> 27 (revolved around decision about <u>recommending</u> vaccine regardless of time frame of vaccine approval).
If you delay or refuse to <u>recommend</u> the COVID-19 vaccine for your patient, which of the following would be the reasons for your decision? Please select all that apply.
(1) I have concerns about side effects or sickness
(2) I believe the vaccine does not work
(3) I am hesitant about vaccine discussion with patients
(4) Patient already had COVID-19
(5) Patient is not in a high-risk or a priority group
(6) Patient has limited contact with high-risk population
(7) I believe COVID-19 is a non-severe or temporary issue
(8) Rapidly changing real world data during pandemic
(9) I believe the ability to social distance and wear a mask provides sufficient protection
(10) Other ________

Question 29: Question will only appear for those who are “somewhat likely,” or “extremely likely,” to get vaccinated in Questions 26 <u>OR</u> 27 (revolved around decision about <u>recommending</u> vaccine regardless of timeframe of vaccine approval)
If you were to <u>recommend</u> the COVID-19 vaccine <u>for your patients</u>, which of the following would be reasons for your decision? Please select all that apply.
(1) Reduce the risk of contracting COVID-19
(2) Avoid transmitting COVID-19 to the patients’ family
(3) Avoid transmitting COVID-19 to the community
(4) Target patients that are at a higher risk for COVID-19 according to CDC criteria
(5) Recommendations of government health agencies (CDC, FDA, NIH, IDSA etc.)
(6) Recommendations of political leaders
(7) Other _________</td>
</tr>
<tr>
<td></td>
<td>General</td>
<td>Please answer Questions 30-33 with the assumption that a COVID-19 vaccine</td>
</tr>
</table>

**Vaccine-specific Clinical information**	Perceived barriers to COVID-19 vaccine<br><br>Adapted from H1N1 Alkuwari et. al Qatar survey study	**had just been approved and deemed safe and effective by the FDA. Please answer with how strongly you agree or disagree with the following statements.**<br><br>**Question 30:**<br>**I would feel protected if I received COVID-19 vaccination**<br>(1) Strongly disagree<br>(2) Disagree<br>(3) Neutral<br>(4) Agree<br>(5) Strongly Agree<br><br>**Question 31:**<br>**I would fear for serious side effects from the COVID-19 vaccine**<br>(1) Strongly disagree<br>(2) Disagree<br>(3) Neutral<br>(4) Agree<br>(5) Strongly agree<br>**Question 32:**<br>**I would doubt or be suspicious about the efficacy of a new COVID-19 vaccine**<br>(1) Strongly disagree<br>(2) Disagree<br>(3) Neutral<br>(4) Agree<br>(5) Strongly agree<br>**Question 33:**<br>**I would worry that the FDA overestimated the safety of the COVID-19 vaccine**<br>(1) Strongly disagree<br>(2) Disagree<br>(3) Neutral<br>(4) Agree<br>(5) Strongly agree
	To gauge what clinical information within the package insert and phase 1/phase 3 clinical studies of a vaccine a pharmacist will prioritize when evaluating a new vaccine<br><br>This focuses on what clinical data pharmacist will prioritize in their vaccine decision-making	**Question 34:**<br>**When asked to make a clinical decision to RECIEVE a new COVID-19 vaccine for YOURSELF, which of the following would you prioritize. Please select your top 3 choices.**<br><br>(1) Indication & dosing schedule<br>(2) Level of antibody titers produced<br>(3) Reduced disease severity and/or transmissibility<br>(4) Mortality reduced<br>(5) Length of protection<br>(6) Percentage of mild side effects<br>(7) Percentage of Grade 2/3/4 side effects<br>(8) Number of participants included in phase 3/4 clinical trials<br>(9) Warnings, Precautions, and contraindications<br>(10) Use in special populations<br>(11) Clinical pharmacology (Mechanism of action)<br>(12) How the vaccine is supplied, stored, and handled<br>(13) Implementation and deployment strategy for mass immunization effort<br>(14) The respective pharmaceutical company's reputation in vaccine development<br><br>**Question 35:**<br>**When asked to make a clinical decision to RECOMMEND a new COVID-19 vaccine for your PATIENTS, which of the following would you prioritize. Please select your top 3 choices.**<br>(1) Indication & dosing schedule<br>(2) Level of antibody titers produced

		(3) Reduced disease severity and/or transmissibility<br>(4) Mortality reduced<br>(5) Length of protection<br>(6) Percentage of mild side effects<br>(7) Percentage of Grade 2/3/4 side effects<br>(8) Number of participants included in phase 3/4 clinical trials<br>(9) Warnings, Precautions, and contraindications<br>(10) Use in special populations<br>(11) Mechanism of action<br>(12) How the vaccine is supplied, stored, and handled<br>(13) Implementation and deployment strategy for mass immunization effort<br>(14) The respective pharmaceutical company's reputation in vaccine development
